# Succession matters: Community shifts in moths over three decades increases multifunctionality in intermediate successional stages

**DOI:** 10.1038/s41598-019-41571-w

**Published:** 2019-04-03

**Authors:** Jan Christian Habel, Andreas H. Segerer, Werner Ulrich, Thomas Schmitt

**Affiliations:** 1 Evolutionary Zoology, University of Salzburg, A-5020 Salzburg, Austria; 2 Bavarian Natural History Collections, Bavarian State Collection of Zoology, D-81247 Munich, Germany; 3 Chair of Ecology and Biogeography, Nicolaus Copernicus University Torun, Lwowska 1, PL-87-100, Toruń, Poland; 4 Senckenberg Deutsches Entomologisches Institut, D-15374 Müncheberg, Germany; 5 Department of Zoology, Institute of Biology, Faculty of Natural Sciences I, Martin-Luther-University Halle-Wittenberg, D-06099 Halle (Saale), Germany; 6 Terrestrial Ecology, Technical University Munich, Hans-Carl-von-Carlowitz-Platz 2, D-85354 Freising, Germany

## Abstract

Species composition strongly depends on time, place and resources. In this context, semi-natural grasslands belong to the most species-rich habitats of Europe, and succession may eventually cause local extinction of typical grassland species, but conversely increase species richness due to habitat diversification. Here, we analyse potential effects of succession of calcareous grasslands on moths. Our studied community, assessed over three decades in south-eastern Germany, comprised >1000 species. We compiled data on the ecology of each of these species, considering the larval and adult stages. We assigned Ellenberg indicator values to each main larval food plant species used by these lepidopterans. Changes in the community means of these indicators were applied to test for possible consequences of the changes in habitat structure and quality. Our data revealed increasing multifunctionality of community structure, higher variability of habitat association over time, the appearance of range expanding species, but also local extinction of various typical grassland moth species. These shifts in species composition mirror effects of succession, which frequently transform previously homogenous semi-natural grasslands into a heterogeneous habitat mosaic.

## Introduction

Evidence for the global loss of species is mainly based on analyses of well-studied, but comparatively species-poor groups of organisms, in particular vertebrates and vascular plants^1,2^. There exists the broad consensus that species richness is negatively influenced primarily by human activities, leading to habitat destruction or deterioration^3^. The habitat quality of the remaining habitats is often affected by habitat fragmentation, increased geographic isolation^4^, and edge effects.

Changing land-use hence is challenging the conservation of biodiversity. While land-use intensification and high productivity farming is directly destroying high-quality habitats by its complete conversion into high productivity fields, the abandonment of traditional low-intensity land-use practices causes succession and is interpreted as another important driver causing species decline^5,6^. Although early successional stages are often beneficial for species’ diversity, loss of biodiversity is often observed for older stages, as e.g. shown for European semi-natural grasslands^7^. A quantification of such environmental changes and its impact on species communities is provided by the concept of multifunctionality^8^. In this context, multifunctionality is defined as the manifold of ecosystem functions realized by a community^9^ and quantifies the performance of various important traits within a single metric^10,11^.

**Figure 1:**
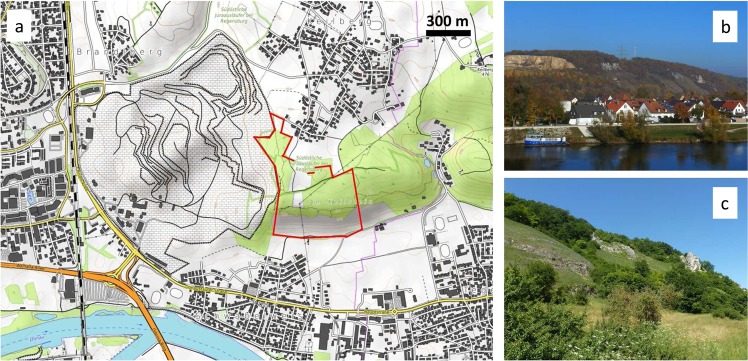
Topographic map of the study area with the approximate border of the investigated area indicated in red (**a**), general aspect of the slopes from the south-east (**b**) and a closer view of the grass- and bushland habitats typical for the site (**c**). The map was reproduced from the OpenTopoMap web site (https://opentopomap.org) under creative common licence CC BY-SA 3.0 (https://creativecommons.org/licenses/by-sa/3.0/legalcode), with subsequent highlighting of the study area. Photographs were taken by AHS.

Recent studies revealed dramatic losses of species and changes in species communities for various groups of insects^12–17^. In this context, lepidopterans represent an excellent model group to study potential responses on environmental changes, as this group consists of a high number of taxa, and is taxonomically as well as ecologically well understood^16,18–20^. However, most studies on lepidopterans have focused on butterflies, but the moths, which are mostly nocturnal, are much more species rich. As a consequence, this important group has been mostly neglected for ecological assessments because data collecting is much more time consuming and taxonomists are much rarer than for the day-active and comparatively species-poor butterflies. In this study, we therefore focus on this group of the mostly nocturnal macro- and micromoths and analyse potential shifts in their species richness and community composition over a period of three decades starting in the late 1980s. Each moth species observed during our survey was classified according its ecological habitat demands and behaviour. Our study area in the vicinity of Regensburg (Bavaria, SE Germany; Fig. 1), which prior to the 1960s had consisted of homogeneous calcareous grasslands, has developed due to on-going strong succession into a habitat mosaic consisting of grassland patches, stony slopes, shrubs and forests. Based on our data, we test the following two hypotheses:

(i) Succession causes a loss of species richness and the local extinction of typical grassland specialist species.
(ii) Succession drives species multifunctionality and the variability of habitat association in species assemblies.

## Results

1,016 moth species with a total of 3,715 records (i.e. species registered within one year) were assessed from 1987 to 2017; due to uneven sampling intensity, the numbers of individuals observed per year were not used for statistical analyses. Our data revealed a significant loss of species over time, with a vanishing of 122 out of the 1,016 species (i.e. a loss of 12%) comparing the species found since the year 2000 and before; most of these species represent specialist species of semi-natural calcareous grassland habitats. However, a total of 411 species was only recorded after the year 2000. Most of these species (257, i.e. 62.5%) are species of forested habitats, forest edges, bushlands or tall-herb communities. About 5% of these newly recorded species (n = 22) belong to the species pool expanding their range over the last few years, and some few (n = 11) belong to groups of sibling species that may have been overlooked in the era before DNA barcoding^16^.

**Figure 2:**
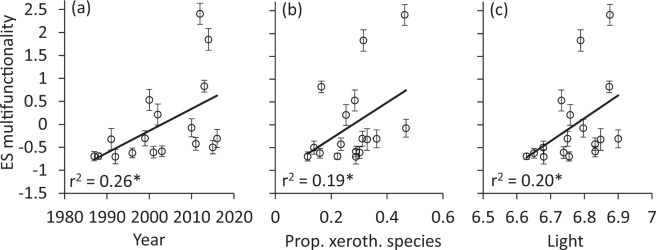
Effect sizes of multifunctionality significantly increased during the study period (**a**), with at higher proportions of xerothermophilic species (**b**), and with rising openness (and subsequently increase of light and temperature) (**c**). Error bars denote randomizations based on 95% confidence limits of the fixed–fixed null model. Permutation significances of the regressions: *P < 0.05.

The M-index of multifunctionality increased over time (Fig. 2a), as well as with the proportion of xerothermophilic moth species, i.e. species preferring rather hot and dry conditions (Fig. 2b). M also increased with rising habitat light regime (Fig. 2c), but was not significantly correlated with humidity, temperature, and nitrogen demands of the larvae assessed by the indicator values of their main food plants (all P > 0.05, not shown). Multifunctionality was highly variable across time (Fig. 2). In 71 of the 136 pairwise comparisons between study years (i.e. 52.2%), annual differences were significant at P < 0.05 (Fig. 2).

**Figure 3:**
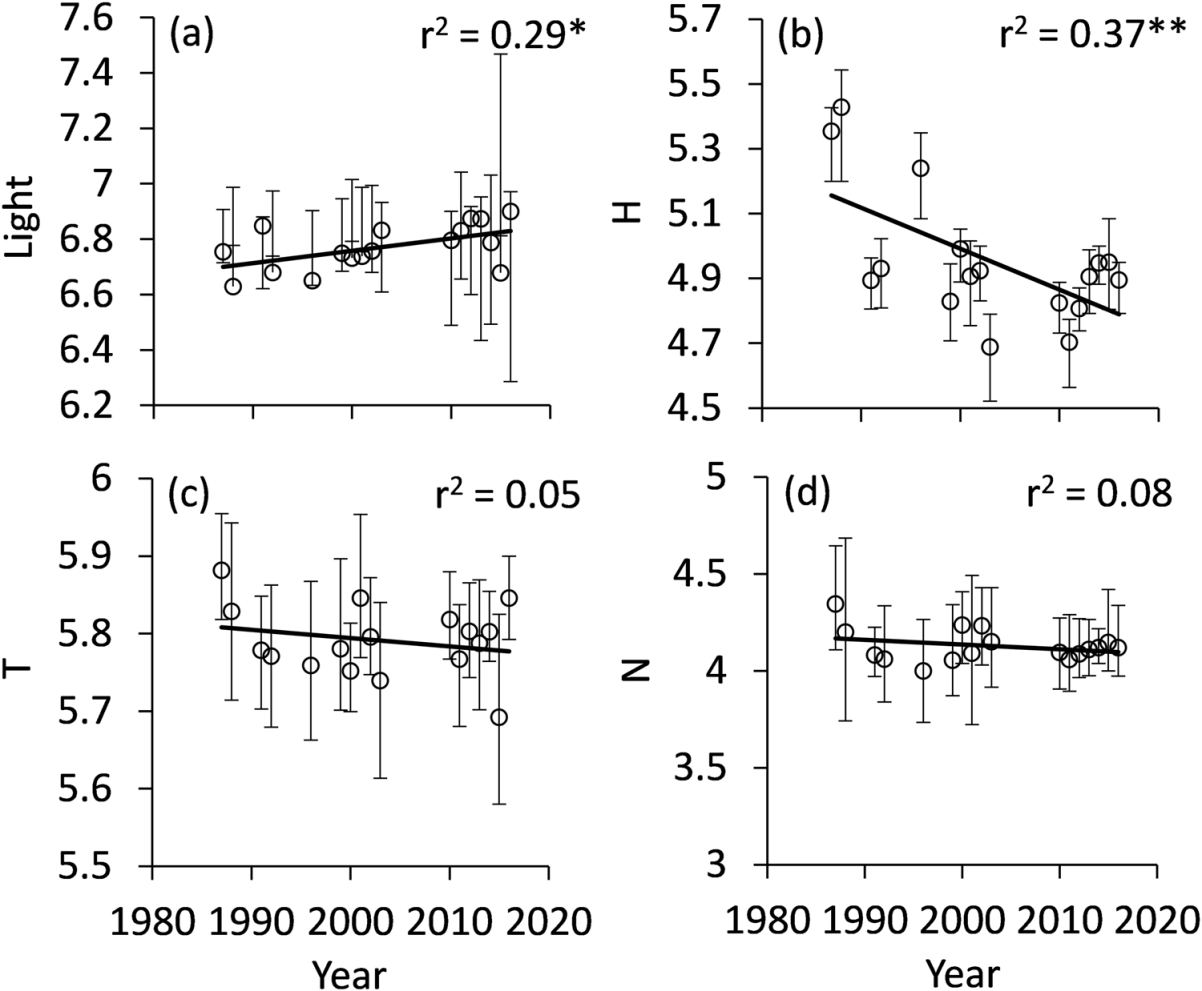
Changes of mean Ellenberg indicator values of the main larval food plants, with respect to light (**a**), humidity (**b**), temperature (**c**), and nitrogen demands (**d**). Permutation significances of the regressions: *P < 0.05; **P < 0.01. Error bars denote bootstrapped lower and upper 95% confidence limits.

**Figure 4:**
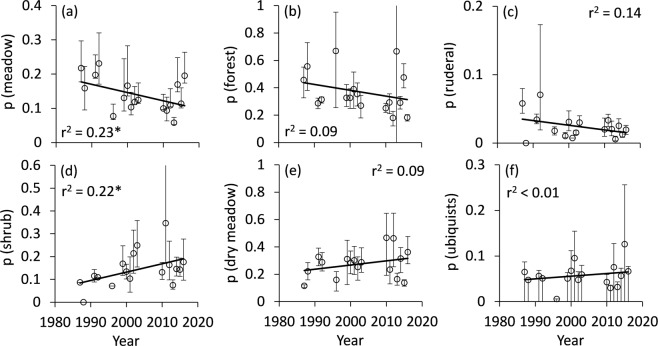
Annual changes in the proportions of moth species associated with meadows/pastures (**a**), forests (**b**), ruderal habitats (**c**), shrubs (**d**), dry meadows (**e**), as well as ubiquitious species (**f**). Error bars denote bootstrapped lower and upper 95% confidence limits. Permutation significances of the regressions: *P < 0.05.

**Figure 5:**
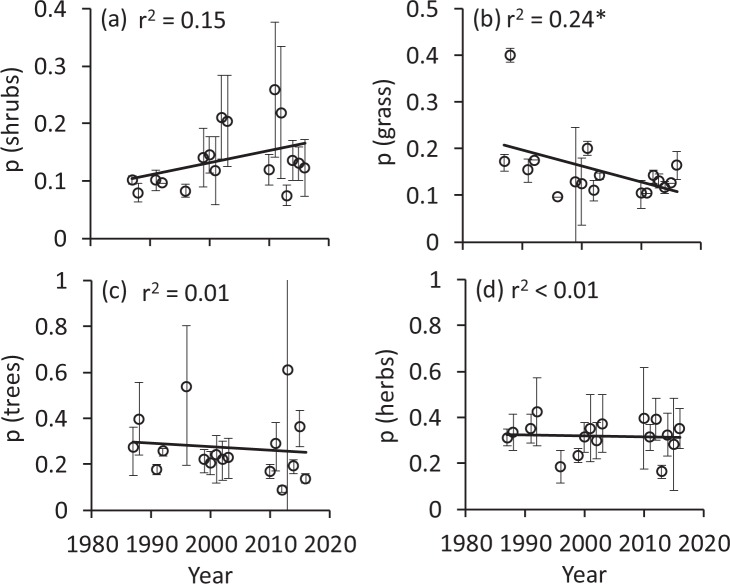
Proportions of larvae associated with shrubs (**a**), grass (**b**), trees (**c**), and herbs (**d**). Error bars denote bootstrapped lower and upper 95% confidence limits. Permutation significances of the regressions: *P < 0.05.

**Figure 6:**
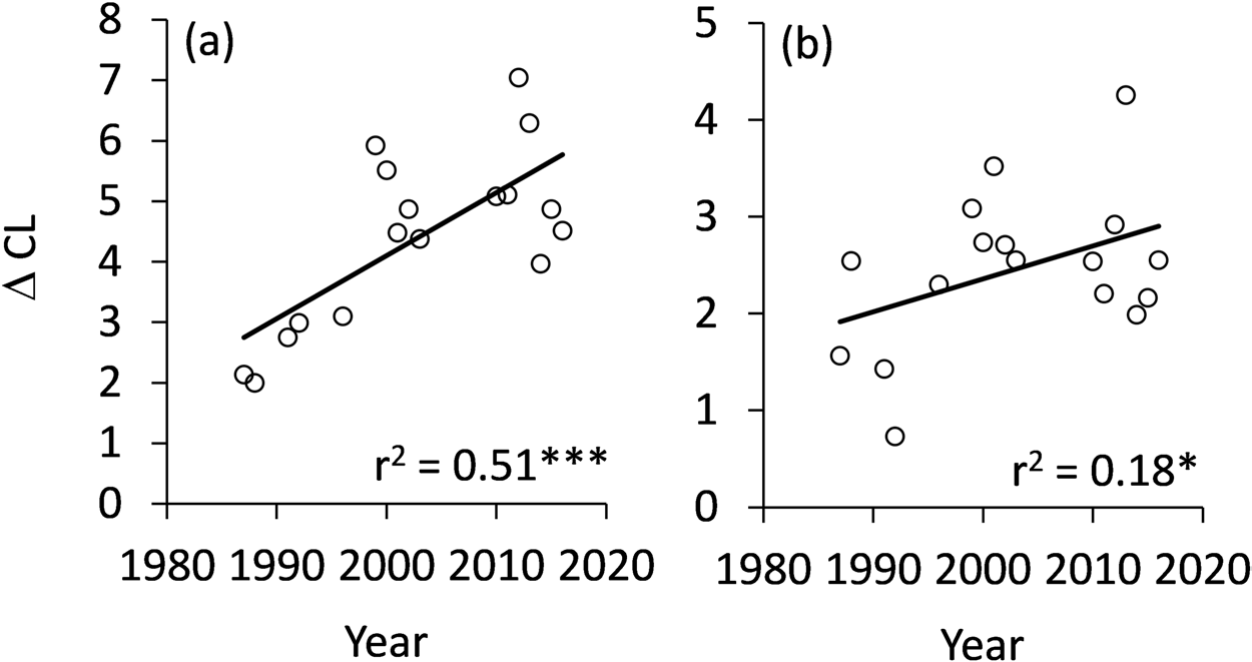
Variability in habitat associations as quantified by the difference Δ between the upper and lower confidence limits CL summed over imaginal habitats (**a**) and larval host plant types (**b**) given in Figs 3 and 4 increased during study time. Permutation significances of the regressions: *P < 0.05, ***P < 0.001.

Following the ecological needs of the main larval food plants (i.e. their Ellenberg indicator values), species associated with open sun exposed habitats increased (Fig. 3a), while those associated with humid habitats (Fig. 3b) decreased. We did neither find temporal trends in temperature (Fig. 3c), nor soil nitrogen availability (Fig. 3d). In line with these changes, we found decreasing proportions of species known to be associated with meadows/pastures (Fig. 4a), forests (Fig. 4b), and ruderal vegetation (Fig. 4c) with only the first being significant; species associated with shrubs (Fig. 4d) and dry meadows (Fig. 4e) increased, but the latter was not significant. We obtained no significant changes for ubiquists (Fig. 4f). The proportion of species with larvae developing on shrubs increased, but the correlation was not significant (Fig. 5a), while those depending on grasses decreased significantly (Fig. 5b). No significant trends were detectable for larvae associated with trees (Fig. 5c) and herbs (Fig. 5d). We found a significant increase in the variability of habitat use (Fig. 6a) and larval plant associations (Fig. 6b), which indicates an increasing variability in species composition over the three studied decades.

## Discussion

We found an increase in species multifunctionality and a higher variability of habitat associations, with an increasing proportion of species relying on shrubs. This is in concordance with the changes in our study area transforming it from a landscape formerly strongly dominated by open grasslands into a diverse mosaic consisting of various habitat types, such as shrubs and forests, ruderal vegetation and grassland patches. At the first glance, this patchy environment of various successional stages today is driving species diversification and is enhancing biodiversity as documented by more than 400 species only recorded after the year 2000. However, ongoing succession in the future may turn it into uniform landscape structures again, consisting of shrubs and trees and not of meadows and pastures. Subsequent losses of species richness would be the inevitable consequence of this process. This trend of biodiversity impoverishment was shown in various ecological studies on succession, showing negative long-term consequences on species richness^6^.

In more detail, most studies analysing temporal trends of species communities and species richness revealed that, today, species communities are frequently dominated by few habitat generalists, while many habitat specialist went extinct^12,13,15,18^. Such a trend was also supported in a recent study on moths^21^. These community modifications can be multicausal. Thus, as demonstrated for spiders, individuals of specialist species either do not find sufficient habitat for their survival, or, even worse, are attracted by ecologically unsuitable habitats representing “ecological traps”^22^. Consequently, our data document a loss of more than 120 species over the last three decades, but did not support a significant decrease of typical grassland species. This might be due to the large number of taxa assessed in this study so that the vanishing of some highly specialised grassland species might not be reflected in an overall significant trend. However, effects of habitat deterioration of semi-natural grasslands are directly detectable by the loss of some characteristic species over the last three decades (e.g. *Depressaria hofmanni*, *Phtheochroa rugosana*, *Pyropteron affinis*, *Triberta helianthemella*, *Cochylimorpha hilarana*, *Eucosma metzneriana*, *Cucullia artemisiae*, *Bucculatrix ratisbonensis*, *Coleophora succursella*, *Eupithecia innotata*). These species were recorded in high densities in former times (1850–2000)^23^, but vanished since then. The larvae of these locally extinct species all depend on specific food plants growing in open, xerothermic and sun-exposed microhabitats, as documented by their Ellenberg values. Today, such habitats are getting scarcer as they become overgrown by successional processes or get lost due to the general eutrophication of the landscapes, which erases the traditional habitats poor in soil nitrogen^13,24^.

Other species (e.g. *Agdistis adactyla*, *Aethes williana*) have been common and widely distributed throughout the area^23^ (and own observations, AHS). Today, they are very rare or even confined to the exposed slopes of the neighbouring stone quarry^17^, which today is the only part of the study area that provides highly disturbed xerothermic stony habitats with scarce vegetation. Such habitats have represented a considerable proportion of the area when it was mostly used as intensive sheep pasture in the past, but are rare today in our study area, and beyound, throughout major parts of open landscapes of Central Europe. This again underlines the importance of such highly disturbed areas as surrogate habitat for high intensity pastures, hence providing important habitats for specialised and endangered taxa.

However, apart from the vanishing and decline of species, we also recorded new taxa not being recorded before the year 2000 (in total 411 species). Some of these species currently expand their geographic distribution ranges, either northwards or eastwards, most probably due to climate change (e.g. *Blastobasis phycidella*, *Catephia alchymista*, *Clavigesta purdeyi*, *Gymnoscelis rufifasciata, Herrichia excelsella, Vitula biviella*^15,16,25^). These species therefore can be seen as the benefiting species from climate change, a consequence already documented since the end of the last century^26,27^. This strongly underlines that increasing temperatures are not only a hazard to biodiversity^28,29^, but also enable the expansion of thermophilic generalists to previously climatically unsuitable areas. However, the majority (63%) of newly recorded species comprises those typical for woodland, bushland, forest edges, and tall forb communities^16^. Their appearance within the last decades obviously reflects the ongoing successional processes and microclimatic changes.

Temporal changes in species compositions in many cases show contrasting trends for moths and butterfly species^30^, and a publication on how both groups react on the conservation of calcareous grasslands revealed that butterflies showed immediate positive reactions while moths did not^20^. In this context, it is not surprising that data on butterflies collected in the same study area and covering a period of nearly 200 years showed strongly diverging results, including severe losses of species during the past three decades and a significant reduction of xerothermophilic species^15^. There arise various non-exclusive explanations leading to these opposite trends obtained for butterflies and moths, but assessed for the same region and identical time period: (i) Differences might arise from different sampling techniques with transect counts for butterflies^31^ and light trapping for the majority of moth species being active during the night, but also day-active species assessed with transect counts. Light trapping also implies the attraction of species from surrounding environments and thus may produce an accumulation of taxa, as for example the attraction of specialist species from the neighbouring stone quarry^32–34^. However, the distance from which moths are attracted to light traps has been shown to be very limited by several studies, hence making this problem of minor relevance^35–38^. Even though, transect counts are more representative for the assessed habitat with its specific conditions. (ii) The occurrence of still many xerothermophilic species might represent an example of a time-lag leading to a final vanishing of these species in the near future^39^: Our data do not consider any details on species’ density; therefore, the current situation might picture a still positive situation, while population densities are already decreasing. (iii) We found a significant loss (12%) of moth species during the study period. However, potential changes of proportional shifts were calculated against a high number of species (>1000). This high reference value of species numbers might blur potential trends so that underlying trends are no more detectable. In contrast, for butterflies, the vanishing of some few grassland habitat specialists already produces a significant loss of this kind of taxa.

Certainly, the intermediate stage of succession studied here has some positive effects on an ecologically diverse group like moths in the short run. However, this stage already shows highly negative effects on groups such as butterflies^15^, which in their majority are species of meadows and their ecotones so that the losses of these species cannot be compensated by different ecological groups as in moths. However, an on-going succession finally will cause the vanishing of most grassland moth species and others of open spaces so that a time-shifted species impoverishment, but in general similar to the one in butterflies, in this group also is a future scenario.

## Methods

Our study area covers roughly 0.35 km^2^ in the north-eastern part of the city of Regensburg, Bavaria, south Germany, and is located around 49.03°N/12.15°E (WGS84) (Fig. 1a). It is a conservation area (“Am Keilstein”, reference number 300.08; “Südöstliche Juraausläufer bei Regensburg”, reference number 300.50) with south-facing slopes along the Danube River and an adjacent limestone quarry. The habitat structures have considerably changed during the past decades: Major proportions of the open grasslands have become overgrown by shrubs and trees in the wake of natural succession, while some remaining grassland patches are kept open by continuous conservation management. Today, the study area is characterised by xerothermic grassland patches, dissected by shrubs, forest and stony slopes in the neighbouring stone quarry (Fig. 1b,c). Semi-natural calcareous grassland patches have been existing continuously in the study area since several centuries and are habitat of a large number of oligotrophic and xerothermophilic plant and animal species^14,25,40^, and thus are partially protected and managed as nature reserve today.

### Data set

Moth species (Macroheterocera and Microlepidoptera, excluding Papilioniodea and Zygaenidae) were assessed by AHS from 1987 until today (March to November) during night using light traps set across the study area and during day along transects. Species identification was performed mainly by AHS, but was also confirmed with DNA barcoding^41^. Data were completed with information from collection records stored in the Zoological State Collection Munich, Germany, as well as from literature^42^. As the intensity of data collection varied among years, we excluded abundance data and reduced our data to a presence-only data set. Years with less than 60 species records were excluded from further analyses as they are not representative for the entire community and hence not suited for a sound statistical analysis.

### Ecology of species

Each moth species was classified according to its ecological demands and behaviour, with data from literature and local observations by AHS. We assigned species into the following groups according to their habitat preference: Calcareous grasslands, meadows/pastures, ruderal vegetation, dwarf-shrubs, woody structures, forests, species living in aquatic ecosystems, bogs, and ubiquitous species. We also classified the species according five larval diet categories: grasses, herbs, shrubs, trees, others. Furthermore, we assessed the food plant species being mainly used by each species in our study area (classification was conducted based on the following literature sources^22,43–47^ and own observations (AHS). We classified each food plant according its biotic and abiotic demands using Ellenberg Indicator values, considering the parameters light, temperature, humidity, and nitrogen^48^. All species specific ecological information is given in Appendix S1.

### Statistics

We calculated the proportions of species being associated with specific habitat types during the imaginal stage, based on species occurrences of all years with at least 60 species recorded. For these years, we calculated proportions of species living in a specific larval habitat according to their larval food plant (classified into trees, shrubs, grasses, herbs). Over all moth species, we extracted Ellenberg indicator values for their respective main larval food plant. Lower and upper one-sided 95% confidence limits were obtained from respective 9,999 bootstrap samples. The means and variances are calculated for each year from matrix randomizations.

We assessed multifunctionality following the approach of Maestre *et al*.^49^ and calculated for each year the M-index being the average of the Z-scores of each Ellenberg based habitat variable, where *x*_i_ denotes the average Ellenberg score in year *i*, and *μ* and *σ* are the mean and standard deviation of these scores, respectively. High values of these variables, particularly of humidity and nitrogen, are linked to increased energy flow and productivity. The M-index is statistically robust and widely used^50^. To account for possible temporal trends and effects of unequal sample sizes on M, we used a null model approach and calculated μ and σ from 200 randomizations of the species × study year presence–absence matrix using the independent swap algorithm that retains observed species numbers per year and numbers of species occurrences among years (fixed–fixed null model)^51^.

We assessed temporal trends in habitat associations using ordinary least squares linear regression. Statistical significance was tested using 9999 permutations of the dependent variable among study years.

## Supplementary information

S1

## References

[1] Pimm SL (2014). The biodiversity of species and their rates of extinction, distribution, and protection. Science.

[2] Ceballos G, Ehrlich PR, Dirzo R (2017). Biological annihilation via the ongoing sixth mass extinction signalled by vertebrate population losses and declines. Proc. Nat. Acad. Sci. USA.

[3] Sala OE (2000). Global biodiversity scenarios for the year 2100. Science.

[4] Fahrig L (2003). Effects of habitat fragmentation on biodiversity. Ann. Rev. Ecol. Evol. Sys..

[5] Huemer P, Tarmann G (2001). Artenvielfalt und Bewirtschaftungsintensität: Problemanalyse am Beispiel der Schmetterlinge auf Wiesen und Weiden Südtirols. Gredleriana.

[6] Peco B, Sánchez AM, Azcárate FM (2006). Abandonment in grazing systems: Consequences for vegetation and soil. Agricult. Ecosys. Env..

[7] Strijker D (2006). Marginal lands in Europe - causes of decline. Basic Appl. Ecol..

[8] Gamfeldt L, Roger F (2017). Revisiting the biodiversity–ecosystem multifunctionality relationship. Nature Ecol. Evol..

[9] Hector A, Bagchi R (2007). Biodiversity and ecosystem multifunctionality. Nature.

[10] Lefcheck JS (2015). Biodiversity enhances ecosystem multifunctionality across trophic levels and habitats. Nature Comm..

[11] Soliveres S (2016). Locally rare species influence grassland ecosystem multifunctionality. Phil. Trans. Roy. Soc. Lond..

[12] Augenstein B, Ulrich W, Habel JC (2012). Directional temporal shifts in community structure of butterflies and ground beetles in fragmented oligotrophic grasslands of Central Europe. Basic Appl. Ecol..

[13] Filz KJ, Engler JO, Stoffels J, Weitzel M, Schmitt T (2013). Missing the target? A critical view on butterfly conservation efforts on calcareous grasslands in south-western Germany. Biodiv. Cons..

[14] Segerer AH (2012). Die physikalisch-geochemischen Grundlagen des planetaren Klimas und die Auswirkungen auf die öffentliche Diskussion - potenzielle Fallstricke für Ökofaunisten. Nachrichtenblatt der bayerischen Entomologen.

[15] Habel JC (2016). Butterfly community shifts over two centuries. Conserv. Biol..

[16] Haslberger A, Segerer AH (2016). Systematische, revidierte und kommentierte Checkliste der Schmetterlinge Bayerns (Insecta: Lepidoptera). Mitteilungen der Münchner Entomologischen Gesellschaft.

[17] Haslberger A, Guggemoos T, Lichtmannecker P, Grünewald T, Segerer AH (2016). Bemerkenswerte Schmetterlingsfunde aus Bayern im Rahmen laufender Projekte zur genetischen Re-Identifizierung heimischer Tierarten (BFB, GBOL) - 7. Beitrag (Insecta: Lepidoptera). Nachrichtenblatt der Bayerischen Entomologen.

[18] Wenzel M, Schmitt T, Weitzel M, Seitz A (2006). The severe decline of butterflies on western German calcareous grasslands during the last 30 years: a conservation problem. Biol. Conserv..

[19] Thomas CD (2000). Dispersal and extinction in fragmented landscapes. Proc. R. Soc. Lond. B.

[20] Rákosy L, Schmitt T (2011). Are butterflies and moths suitable ecological indicator systems for restoration measures of semi-natural calcareous grassland habitats? *Ecol*. Ind..

[21] Mangels J, Fiedler K, Schneider FD, Blüthgen N (2017). Diversity and trait composition of moths respond to landuse intensification in grasslands: generalists replace specialists. Biodivers. Conserv..

[22] Mason LD, Bateman PW, Wardell-Johnson GW (2018). The pitfalls of short-range endemism: high vulnerability to ecological and landscape traps. PeerJ.

[23] Hofmann, O. & Herrich-Schäffer, G. A. W. Die Lepidopteren-Fauna der Regensburger Umgegend. *Korrespondenz-Blatt des zoologisch-mineralogischen Vereines in Regensburg* 8 101–109, 113–128, 129–144, 167–176, 177–190 (1854); 9 57–72, 73–88, 133–136, 137–149 (1855).

[24] Bobbink R, Hornung M, Roelofs JGM (1998). The effects of air-borne nitrogen pollutants on species diversity in natural and semi-natural European vegetation. J. Ecol..

[25] Segerer AH, Lichtmannecker P, Grünewald T, Lohberger E (2013). Aktuelle Vorkommen einiger wenig bekannter Schmetterlingsarten in Deutschland (Lepidoptera, Nepticulidae, Gracillariidae, Gelechiidae, Tortricidae, Crambidae). Entomol. Nachr. Ber..

[26] Warren MS (2001). Rapid responses of British butterflies to opposing forces of climate and habitat change. Nature.

[27] Chen I-C, Hill JK, Ohlemüller R, Roy DB, Thomas CD (2011). Rapid range shifts of species associated with high levels of climate warming. Science.

[28] Settele J (2008). Climatic risk atlas of European butterflies. BioRisk.

[29] Schmitt T, Habel JC, Rödder D, Louy D (2014). Effects of recent and past climatic shifts on the genetic structure of the high mountain Yellow‐spotted ringlet butterfly *Erebia manto* (Lepidoptera, Satyrinae): a conservation problem. Global Change Biol..

[30] Ricketts TH, Daily GC, Ehrlich PR (2002). Does butterfly diversity predict moth diversity? Testing a popular indicator taxon at local scales. Conserv. Biol..

[31] Pollard, E. & Yates, T. J. Monitoring butterflies for ecology and conservation - The British Butterfly Monitoring Scheme. Chapman & Hall, London. (1993).

[32] Truxa C, Fiedler K (2012). Attraction to light - from how far do moths (Lepidoptera) return to weak artificial sources of light?. Europ. J. Entomol..

[33] Merckx T (2012). Conserving threatened Lepidoptera: towards an effective woodland management policy in landscapes under intense human land-use. Biol. Conserv..

[34] Merckx T, Slade EM (2014). Macro-moth families differ in their attraction to light: implications for lighttrap monitoring programmes. Insect Conserv. Divers..

[35] Baker RR, Sadovy Y (1978). The distance and nature of the light trap response of moths. Nature.

[36] Wirooks, L. Die ökologische Aussagekraft des Lichtfangs: Eine Studie zur Habitatanbindung und kleinräumigen Verteilung von Nachtfaltern und ihren Raupen. Havixbeck-Hohenholte: Wolf & Kreuels, 302 pp. (2005).

[37] Beck J, Linsenmair KE (2006). Feasibility of light-trapping in community research on moths: Attraction radius of light, completeness of samples, nightly flight times and seasonality of Southeast-Asian hawkmoths (Lepidoptera: Sphingidae). J. Res. Lepid..

[38] Truxa C, Fiedler K (2012). Attraction to light – from how far do moths (Lepidoptera) return to weak artificial sources of light?. Europ. J. Entomol..

[39] Krauss J (2010). Habitat fragmentation causes immediate and time-delayed biodiversity loss at different trophic levels. Ecol. Letters.

[40] Bernhard E, Owen M (1990). Stadtbiotopkartierung Regensburg unter besonderer Berücksichtigung der Pflanzen- und Tierwelt auf Kalk am Beispiel des Gebietes Brandlberg und Keilberg mit Keilstein und Spitalholz, pp. 64–68. – In: Bayerisches Landesamt für Umweltschutz (Hrsg.): Schutzwürdige Biotope in Bayern (2). Stadtbiotopkartierung I. Schriftenreihe des Bayerischen Landesamtes für Umweltschutz.

[41] Hausmann A (2011). Now DNA-barcoded: the butterflies and larger moth of Germany (Lepidoptera: Rhopalocera, Macroheterocera). Spixiana.

[42] Segerer AH (1997). Verifikation älterer und fraglicher Regensburger Lepidopterenmeldungen. Beiträge zur bayerischen Entomofaunistik.

[43] Schütze, K. T. Die Biologie der Kleinschmetterlinge unter besonderer Berücksichtigung ihrer Nährpflanzen und Erscheinungszeiten. Frankfurt am Main: *Verlag des Internationalen Entomologischen Vereins E. V*., 235 pp. (1931).

[44] Hering, E. M. Biology of the leaf miners. Dr. W. Junk, ‘s-Gravenhage, 420 pp. (1951).

[45] Scoble, M. J. The Lepidoptera. Form, Function and Diversity. Oxford University Press, Oxford, xi + 404 pp. (1992).

[46] Pröse H, Segerer AH, Kolbeck K (2004). Rote Liste gefährdeter Kleinschmetterlinge (Lepidoptera: Microlepidoptera) Bayerns. Schriftenreihe des Bayerischen Landesamtes für Umweltschutz.

[47] Huemer, P. ‘Ausgeflattert’. Der stille Tod der österreichischen Schmetterlinge. Blühendes Österreich - REWE International gemeinnützige Privatstiftung & Umweltschutzorganisation GLOBAL 2000/Friends of the Earth Austria (eds.), 36 pp. (2016).

[48] Ellenberg, H. Zeigerwerte der Gefäßpflanzen Mitteleuropas. 3rd edition. Göttingen: Scripta Geobotanica, Erich Göltze (1992).

[49] Maestre FT (2012). Plant species richness and ecosystem multifunctionality in global drylands. Science.

[50] Byrnes JEK (2014). Investigating the relationship between biodiversity and ecosystem multifunctionality: challenges and solutions. Methods Ecol. Evol..

[51] Gotelli NJ (2000). Null model analysis of species co-occurrence patterns. Ecology.

